# Delineation of plant caleosin residues critical for functional divergence, positive selection and coevolution

**DOI:** 10.1186/1471-2148-14-124

**Published:** 2014-06-09

**Authors:** Wanlu Song, Yajuan Qin, Yan Zhu, Guangjun Yin, Ningning Wu, Yaxuan Li, Yingkao Hu

**Affiliations:** 1 College of Life Sciences, Capital Normal University, Beijing 100048, China

## Abstract

**Background:**

The caleosin genes encode proteins with a single conserved EF hand calcium-binding domain and comprise small gene families found in a wide range of plant species. These proteins may be involved in many cellular and biological processes coupled closely to the synthesis, degradation, or stability of oil bodies. Although previous studies of this protein family have been reported for *Arabidopsis* and other species, understanding of the evolution of the caleosin gene family in plants remains inadequate.

**Results:**

In this study, comparative genomic analysis was performed to investigate the phylogenetic relationships, evolutionary history, functional divergence, positive selection, and coevolution of caleosins. First, 84 caleosin genes were identified from five main lineages that included 15 species. Phylogenetic analysis placed these caleosins into five distinct subfamilies (sub I–V), including two subfamilies that have not been previously identified. Among these subfamilies, sub II coincided with the distinct P-caleosin isoform recently identified in the pollen oil bodies of lily; caleosin genes from the same lineage tended to be clustered together in the phylogenetic tree. A special motif was determined to be related with the classification of caleosins, which may have resulted from a deletion in sub I and sub III occurring after the evolutionary divergence of monocot and dicot species. Additionally, several segmentally and tandem-duplicated gene pairs were identified from seven species, and further analysis revealed that caleosins of different species did not share a common expansion model. The ages of each pair of duplications were calculated, and most were consistent with the time of genome-wide duplication events in each species. Functional divergence analysis showed that changes in functional constraints have occurred between subfamilies I/IV, II/IV, and II/V, and some critical amino acid sites were identified during the functional divergence. Additional analyses revealed that caleosins were under positive selection during evolution, and seven candidate amino acid sites (70R, 74G, 88 L, 89G, 100 K, 106A, 107S) for positive selection were identified. Interestingly, the critical amino acid residues of functional divergence and positive selection were mainly located in C-terminal domain. Finally, three groups of coevolved amino acid sites were identified. Among these coevolved sites, seven from group 2 were located in the Ca^2+^-binding region of crucial importance.

**Conclusion:**

In this study, the evolutionary and expansion patterns of the caleosin gene family were predicted, and a series of amino acid sites relevant to their functional divergence, adaptive evolution, and coevolution were identified. These findings provide data to facilitate further functional analysis of caleosin gene families in the plant lineage.

## Background

Lipids in plant seeds are stored in specialized organelles termed oil bodies (OBs) to serve as an energy source for germination [1]. Seed OBs are composed of a core of neutral lipids, mainly triacylglycerols (TAGs), surrounded by a phospholipid (PL) monolayer embedded with integral proteins [2]. Only a limited number of proteins are specifically associated with OBs; these proteins belong to three major classes comprising structural proteins (oleosins and caleosins), enzymes (e.g., steroleosins and lipases), and minor proteins (e.g., aquaporins) [3]. To date, oleosins, caleosins, and steroleosins have been identified in OBs of various plant species, including *Arabidopsis*, *Sorghum bicolor*, and *Zea mays*[4,5]. The most abundant structural proteins in plants are oleosins, which are basic proteins unique to plants and found in various organs [1,6]. Oleosins have been demonstrated to play roles in the stability, synthesis, and metabolism of OBs [7].

Caleosin genes are widely distributed in true fungi, unicellular microalgae, and higher plants [8]. The first caleosin sequence was cloned and characterized in rice seeds germinating in response to abscisic acid treatment by Fradsen et al. in 1996 [9]. Subsequently, Caleosin was first identified to comprised a calcium-binding motif, and thus tentatively named caleosin by Chen et al. [4], who also proposed a structural model for the protein based on a hydropathy plot and analysis of its secondary structure. The N-terminal hydrophilic domain of caleosin consists of an EF-hand calcium-binding motif of 28 residues including an invariable glycine residue as a structural turning point and five conserved oxygen-containing residues as calcium-binding ligands [4,10]. The central hydrophobic domain of caleosin contains an amphipathic α-helix and an anchoring region. This amphipathic α-helix is assumed to be located at the interface between hydrophobic and hydrophilic environments while the anchoring region is predicted to contain a pair of antiparallel β-strands connected with a proline-knot motif. The C-terminal hydrophilic domain of the protein contains several potential phosphorylation sites [10-12].

Caleosin comprises a calcium-binding motif and several potential phosphorylation sites, that is, well-known candidates involved in signal transduction, and thus may possess biological function(s) in addition to its structural role for the stability of oil bodies [13]. According to the characterization of two independent insertion mutants lacking caleosin, it was proposed that caleosin might play a role in the degradation of storage lipids in oil bodies by inducing the interaction of oil bodies with vacuoles during germination [14]. Putative interaction between oil bodies and vacuoles has also been observed in pollen cells after germination under electron microscopy; and the pollen oil bodies appeared to be surrounded by tubular membrane structures and encapsulated in vacuoles after germination [15,16]. Caleosin isoforms or caleosin-like proteins are not only localized in oil bodies but have also been found as membrane-bound proteins in other subcellular fractions, such as the microsomal membrane; moreover, they were demonstrated to possess different biological functions, such as peroxygenase activity in biotic and abiotic stress responses in their phosphorylated forms [17-19].

Considering the economic significance of oil seeds, caleosin, as one of the primary OB-associated proteins, has been examined extensively [20,21]. However, the evolution of caleosins has not been studied in all plant lineages; a comprehensive comparative genome study would improve understanding of the evolution and function of the caleosin family. In the present work, we identified all caleosin gene families in 15 plant species, representing the major plant lineages with available genome sequences. We then performed phylogenetic analysis, exon/intron structural analysis, and motif analysis to trace the evolutionary history of the caleosin family in plants, and detected segmental duplication and tandem duplication events to gain insight into possible mechanisms for the expansion of caleosin gene families. Analysis of functional divergence using bioinformatics software suggested that changes in selective constraints and amino acid properties occurred after gene duplication, which led to subfamily-specific functional evolution after the diversification of caleosins. Positive selected sites and coevolved sites were also predicted, and finally, three types of critical amino acid sites were proposed.

## Results

### Identification of caleosin genes

To explore the origin and evolutionary history of the caleosin gene family, we identified caleosin genes from 15 species representing the five major plant lineages: the green algae *Chlamydomonas reinhardtii* and *Volvox carteri*; the moss *Physcomitrella patens*; the lycophyte *Selaginella moellendorffii*; the monocotyledonous angiosperms *Brachypodium distachyon*, *Oryza sativa*, *Setaria italica*, *Zea mays*, and *Sorghum bicolor*; and the dicotyledonous angiosperms *Arabidopsis thaliana*, *Citrus sinensis*, *Glycine max*, *Populus trichocarpa*, *Brassica rapa*, and *Cucumis sativus*. We retrieved the available caleosin or caleosin-like genes using the Phytozome, JGI, TAIR, RAP, and BRAD databases, and BLASTP search results identified 101 caleosin homologue genes that encode proteins containing the caleosin domain. The search results were further examined using SMART and PFAM (PF05042) to confirm the presence of the conserved caleosin domain. Finally, we identified 84 caleosin genes from the above 15 species representing five major lineages (Additional file 1 and Additional file 2).

### Phylogenetic relationships and evolution of the caleosin gene family

**Figure 1:**
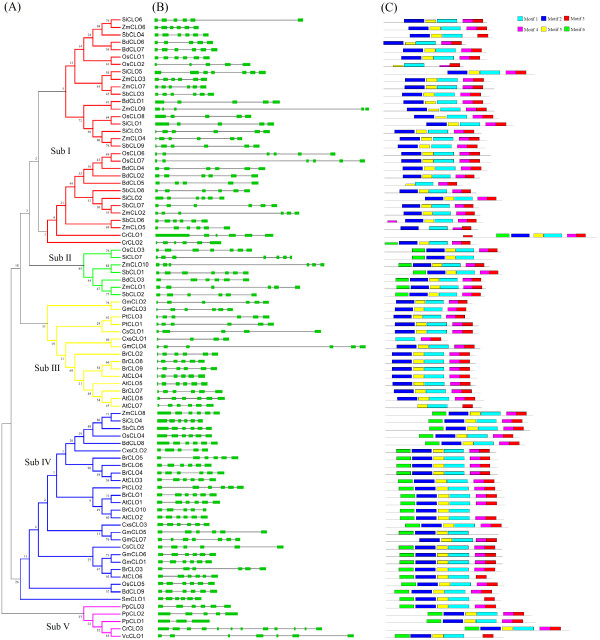
**Phylogenetic relationships, exon-intron structure, and motif structures of caleosin genes. (A)** The neighbor-joining (N-J) phylogenetic tree was constructed based on a complete protein sequence alignment of 84 caleosin genes identified using Clustal X and MEGA5. Numbers at the nodes represent bootstrap support (1000 replicates). The color of subclades indicates the five corresponding gene subfamilies. **(B)** Exon-intron structures of the caleosin genes. Boxes: exons; lines: introns. The lengths of boxes and lines are scaled based on gene length. **(C)** MEME motif search results. Conserved motifs are indicated in numbered color boxes.

To investigate the evolutionary relationships of caleosins among various plant species, the 84 identified protein sequences were used to execute multiple sequence alignment using Clustal X [22]. Based on this alignment, a rooted neighbor-joining (N-J) phylogenetic tree was constructed from the full-length protein sequences using MEGA5 [23]. To further confirm the topology of phylogenetic tree, a maximum likelihood (ML) phylogenetic tree was constructed, which showed similar topology to the N-J tree with only minor modifications (data not shown). According to the topology and the deep duplication nodes of caleosin paralogs in the N-J tree, the caleosin gene families could be divided into five well-conserved subfamilies (Figure 1A, Additional file 3). We numbered these subfamilies sub I, sub II, sub III, sub IV, and sub V in the phylogenetic tree.

Our analysis demonstrated that caleosin genes from the same lineage tended to be clustered together in the phylogenetic tree. Of the five subfamilies, sub V was only present in lower land plants (i.e., green algae and mosses), while subs I–IV were only present in higher land plants (i.e., angiosperms) with three exceptions (*CrCLO1* and *CrCLO2* were located in sub I, and *SmCLO1* was in sub IV). Moreover, subs I and II were found exclusively in monocots (Figure 1A), while sub III was only found in dicots. These observations indicated that the caleosin genes shared a common ancestor before the divergence between lower and higher land plants. We propose an evolutionary pattern for caleosins based on the phylogenetic tree: first, all caleosins shared a common ancestor and the lower plants (sub V) were the first to diverge. Sub IV included both monocots and dicots, which indicated that this subfamily diverged before the monocot-dicot split approximately 200 MYA. The subsequent divergence between subs I, II, and III was thought to have occurred after the monocot-dicot split because all members of subs I and sub II were monocots while members of sub IV were exclusively dicots.

**Figure 2:**
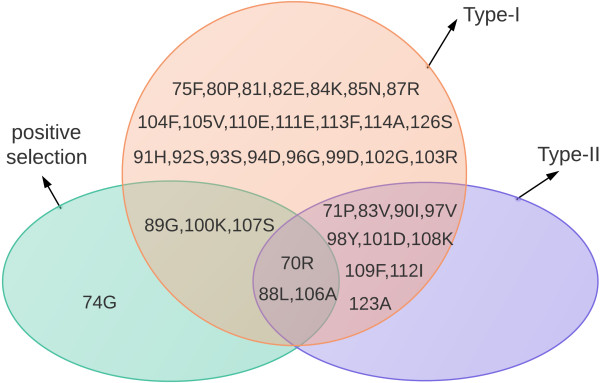
**Venn diagram of Type-I and Type-II amino acid sites related to functional divergence, and positively selected sites.** All sites are positioned on the reference sequence (*AtCLO*5) based on sequence alignment.

We conducted an analysis of the exon-intron structure of individual caleosin genes in all plant lineages using the online Gene Structure Display Server [24] to gain insight into possible mechanisms for the expansion of the multiple gene families. A detailed illustration of the relative length of introns and of conservation of the corresponding exon sequence within each caleosin paralog is provided in Figure 2B. Genes in the same subfamily had similar exon-intron structures. The number of introns did not differ significantly among the caleosin genes and ranged from four to six with the following exceptions: *CsCLO1* from sub III contained three introns; *PpCLO1* from sub V had two introns, and *CrCLO3* from sub V contained 11 introns.

A MEME (Multiple Em for Motif Elicitation) search [25] for conserved protein motifs flanking the caleosin genes was conducted (Figure 1C) to determine possible mechanisms for the structural evolution of caleosin genes. Six motifs were detected and proteins in the same subfamily shared similar numbers and patterns of conserved motifs, The sequence logos for each motif, containing stacks of letters at each position, are shown in Additional file 4. Interestingly, the distribution of Motif 6 revealed obvious regularities that corresponded closely to the phylogenetic relationships: Motif 6 was absent from subs I and III, while all members of subs II, IV, and V, except for *GmCLO7*, contained this motif. Moreover, we observed that caleosin protein sequences from the same species were divided into different subfamilies: the monocot members were separated in sub III and sub IV, while the dicot members were separated in subs I, II, and IV. Multiple alignment of each species was performed to explore the possible causes of this arrangement, and the results are shown in in Additional files 5 and 6 for dicots and monocots, respectively. In the figures, proteins from subs I–IV are colored red, green, yellow, and blue, respectively. As may be seen, the presence or absence of motif 6 is strongly associated with the separation of members into different subfamilies. The distribution of Motif 6 was also consistent with the evolutionary pattern of caleosin that we proposed from the phylogenetic analysis. We infer from these combined data that the ancestor of caleosins may have contained Motif 6 and that all members of sub V still contained this motif after the first divergence, as Motif 6 was conserved in both lower plants and angiosperms. Subsequently, after the second divergence before the monocot-dicot split, Motif 6 was conserved in all sub-IV members except for *GmCLO7*. However, during the divergence between subs I-III, only the members of sub II retained Motif 6.

### Expansion analysis of the caleosin gene family

As is known, duplication at the gene and genome levels is a widespread process that contributes to the evolution of biological novelty [26]. Here, we analyzed segmental and tandem duplications of caleosin genes to investigate the main expansion pathways of caleosins and the differences in these genes between monocots and dicots. We identified 12 pairs of segmentally duplicated genes (Table 1) and nine pairs of tandem duplicated genes (Table 2). To estimate the approximate ages of the duplication events, synonymous base substitution rates (*Ks* values) and four-fold degenerate transversion site (4DTv) distances were estimated as a proxy for time.

**Table 1:** Estimates of the dates for the segmental duplication events of caleosin gene family

**Gene pairs**		**KS (mean ± s.d.)**	**Estimated time (mya)**	**GWD (mya)**	**References**
*OsClo2*	*OsClo6*	0.455 ± 0.105	35.0	30-40	Vandepoele et al., 2003 [27]
*GmClo1*	*GmClo6*	0.169 ± 0.072	13.9	13, 59	Schmutz et al., 2010 [28]
*GmClo5*	*GmClo7*	0.163 ± 0.055	13.4		
*AtClo8*	*AtClo4*	0.712 ± 0.182	23.7	28-48	Ermolaeva et al., 2003 [32]
*AtClo1*	*AtClo2*	0.712 ± 0.112	23.7		
*PtClo1*	*PtClo3*	0.348 ± 0.144	19.1	8-13	Tuskan et al., 2006 [34]
*BrClo1*	*BrClo10*	0.810 ± 0.048	28.9	13-17	Town et al., 2006 [36]
*BrClo2*	*BrClo4*	0.363 ± 0.798	12.9		
*BrClo3*	*BrClo9*	0.453 ± 0.204	16.2		
*BrClo4*	*BrClo5*	0.753 ± 0.059	26.9		
*BrClo4*	*BrClo6*	0.400 ± 0.160	14.3		
*BrClo8*	*BrClo9*	0.353 ± 0.095	12.6		

**Table 2:** 4DTv distance between paralogous genes

**Species**	**Gene pairs**		**D**_**4DTv **_**value**
*Brachypodium distachyon*	*BdClo4*	*BdClo5*	0.399
*Brachypodium distachyon*	*BdClo6*	*BdClo7*	0.414
*Sorghum bicolor*	*SbClo1*	*SbClo2*	0.206
*Sorghum bicolor*	*SbClo7*	*SbClo8*	0.072
*Oryza sativa*	*OsClo1*	*OsClo2*	0.229
*Oryza sativa*	*OsClo6*	*OsClo7*	0.039
*Arabidopsis thalina*	*AtClo8*	*AtClo7*	0.235
*Arabidopsis thalina*	*AtClo4*	*AtClo5*	0.138
*Glycine max*	*GmClo2*	*GmClo3*	0.189

Our results showed that caleosin genes from different species did not share a common expansion model. Only tandem duplications were identified in the monocots *Brachypodium* and *Sorghum*; only segmental duplications were identified in dicots *Populus* and *Brassica*; and both types of duplications were identified in rice, *Arabidopsis*, and soybean (Additional file 7). Moreover, no segmental duplication was identified in sub II and no tandem duplication identified in sub IV, while subs I and III included both types of duplications, suggesting that different subfamilies had different expansion pathways.

Synonymous base substitution rates (*Ks* values) were estimated to calculate the approximate ages of segmental duplication events (Table 1). The one pair occurring in monocots, *OsCLO2*/*OsCLO6* in rice, was predicted to have diverged about 35 MYA during the genome-wide duplication (GWD) event [27], indicating that this gene pair was conserved during evolution after the GWD of rice. Previous studies have demonstrated that the soybean genome was complicated by at least two rounds of GWD at about 13 and 59 MYA [28,29]; thus, it is likely that the pairs *GmCLO1*/*GmCLO6* and *GmCLO5*/*GmCLO7* were conserved after the second ancient large-scale duplication about 13 MYA. Analysis of *Arabidopsis* caleosin segmentally duplicated gene pairs (*AtCLO8*/*AtCLO4* and *AtCLO1*/*AtCLO2*) suggested that the timing of their duplication (approximately 23.7 MYA) was consistent with the emergence of crucifers between 24–40 MYA [30,31] but after its GWD, which occurred approximately 28–48 MYA [32]. The GWD of poplar is thought to have occurred about 8–13 MYA [33] but segmental duplications of poplar caleosin genes were estimated to have occurred earlier, about 19.1 MYA. Despite similar magnitudes of GWD, *Arabidopsis* contained more caleosin genes than poplar (eight and three, respectively), probably because the poplar genome subsequently experienced a high level of gene loss [34]. Among the dicots, *Brassica rapa* showed the largest number of segmental duplications (six), four of which corresponded to a whole-genome triplication event that is thought to have occurred between 13 and 17 MYA [35,36]. We propose that the identified segmentally duplicated gene pairs were mostly conserved after the large-scale duplication event of each species during its evolution. In addition, the two genes of each duplicated pair belonged to the same subfamilies, which suggested that they had not undergone evolutionary divergence after duplication.

Genetic distance-transversion rates at 4DTv of tandem-duplicated caleosin gene pairs were calculated using the PAML software package [37]; 4DTv distance ranged from zero for recently duplicated peptides to ~0.5 for paralogs with an ancient evolutionary past. Among the nine tandem-duplicated pairs, only two (*SbCLO7*/*SbCLO8*, *OsCLO6*/*OsCLO7*) appeared to have occurred in the recent past (4DTv values <0.1), while the others were conserved from more ancient times (Table 2).

### Functional divergence after gene duplication

Two types of functional divergence (Type-I and Type-II) between gene clusters of the caleosin subfamily were estimated using DIVERGE2 [38], which evaluates shifts in evolutionary rate and altered amino acid properties after gene duplication. Type-I functional divergence refers to evolutionary processes resulting in site-specific rate shifts after gene duplication, while Type-II results in site-specific property shifts. Each of the 81 caleosin proteins of the five subfamilies were used as input files in DIVERGE, and the estimates were based on the multiple amino acid sequence alignments of caleosin proteins from any two subfamilies. The coefficients of Type-I functional divergence between subfamily pairs I/IV, II/IV, and II/V were statistically significant (θ > 0; likelihood ratio test statistic > 4.96; p < 0.01), indicating that significantly different site-specific shifts in evolutionary rate may have taken place at certain amino acid sites between these pairs. On the other hand, there was evidence of Type-II functional divergence between four subfamily pairs (I/IV, II/III, II/IV, and II/V), indicating a radical shift in amino acid properties (Table 3).

**Table 3:** Functional divergence between groups of the caleosin subfamily

**Group1**	**Group2**	**Type-I**				**Type-II**	
		**θ**_**I**_ **± sec**	**LRT**	**Q**_**k**_ **> 0.8**	**Q**_**k**_ **> 0.9**	**θ**_**II**_ **± sec**	**Q**_**k**_ **> 0.8**
I	II	0.418 ± 0.188	4.96*	2	0	-0.078 ± 0.449	0
I	III	0.532 ± 0.477	1.25	0	0	-0.278 ± 0.436	0
I	IV	0.914 ± 0.156	34.4**	36	24	0.054 ± 0.448	7
I	V	0.418 ± 0.219	3.64	1	1	-0.343 ± 0.459	0
II	III	0.386 ± 0.332	1.36	0	0	0.112 ± 0.241	8
II	IV	0.993 ± 0.173	32.8**	38	38	0.088 ± 0.342	8
II	V	0.967 ± 0.316	9.39**	38	38	0.027 ± 0.283	6
III	IV	0.001 ± 0.022	0.00	0	0	-0.192 ± 0.324	0
III	V	0.253 ± 0.287	0.78	0	0	-0.212 ± 0.245	0
IV	V	0.242 ± 0.205	1.40	0	0	-0.163 ± 0.339	0

Note: θ_I_ and θ_II,_ the coefficients of Type-I and Type-II functional divergence between two gene clusters; LRT, Likelihood Ratio Statistic, for p < 0.05 was marked by *p < 0.01 was marked by **Q_k,_ posterior probability. Large Q_k_ value indicates a high possibility that the functional constraint (or the evolutionary rate) or the physicochemical properties of a given amino acid site is different between two clusters.

To identify critical amino acid sites that may be responsible for functional divergence between caleosin subfamilies, the posterior probability (*Qk*) of divergence was determined for each site. Large values of *Qk* indicate a high probability that the evolutionary rate or physiochemical amino acid properties of a site differ between two clusters. DIVERGE2 thus identified critical amino acid sites that are highly relevant to functional divergence (Table 3). To greatly reduce false positives, values of *Qk* > 0.8, p < 0.01 were empirically used as a cutoff for identifying Type-I and Type-II functional-divergence-related residues between gene subfamilies. Critical amino acid sites were identified in five groups of caleosin subfamilies for the analysis of Type-I functional divergence. Only two sites were identified between subs I/II, and one site between subs I/V. In contrast, the numbers of amino acid sites identified between subs I/IV, II/IV, and II/V were 36, 38, and 38, respectively. Fewer sites were identified as responsible for Type-II than for Type-I functional divergence, with 7, 8, 8, and 6 sites between subs I/IV, II/III, II/IV, and II/V, respectively (Table 3, Additional file 8). Interestingly, all of the Type-II sites for each subfamily group belonged to the corresponding Type-I sites. In other words, all of the detected Type-II sites of functional divergence that underwent site-specific property shifts also underwent site-specific rate shifts (i.e., Type-I functional divergence) (Figure 2, Additional file 8).

**Figure 3:**
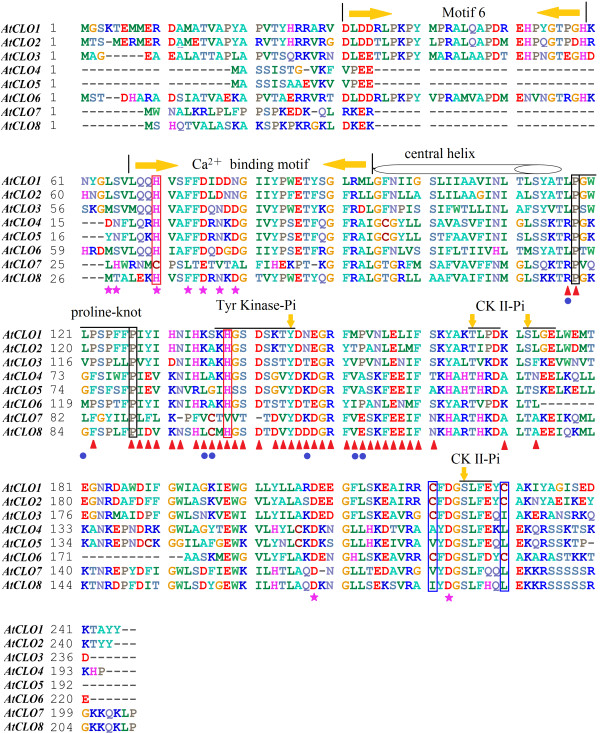
**Multiple sequence alignment of *Arabidopsis *caleosin protein sequences.** The positions of the motif 6, a calcium-binding motif, a proline knot-like motif, and four phosphorylation sites (one tyrosine kinase and three casein kinase II phosphorylation sites) are indicated on the tops of the sequences. The two invariable proline residues in the proline knot-like motif are labeled by black boxes, two His sites and two Cys sites of dissulfide bridge are highlighted by red and blue frames, respectively. The consensus sequence of the phosphorylation sites are marked by upper lines with the potential phosphorylated residue pointed by arrows. The critical amino acid sites of functional divergence, adaptive selection and coevolution are marked by red triangles, blue circles, and purple stars, respectively.

Additional analyses were performed to investigate the critical amino acid sites responsible for functional divergence. Their relationships to four motifs supposed to be functionally important (Ca^2+^ binding motif, Proline-knot, Tyr kinase-Pi, and CKII-Pi) [4] were identified: three sites coincided with the proline-knot motif, eight sites coincided with Tyr kinase-Pi, and two sites were located in the CKII-Pi motif (Figure 3, Additional file 8). The majority of the amino acid sites (29 of 38) were distributed in the C-terminal domain of caleosin. While the functions of these sites need to be experimentally verified. Thus, the results of the functional divergence analysis suggest that, because of the different evolutionary rates predicted at some amino acid sites, the caleosin genes may be significantly divergent from each other in their functions. Mutations in amino acids may have caused the caleosin gene family to evolve new functions after divergence, and hence, functional divergence might reflect the existence of long-term selective pressures.

### Variable selective pressures among amino acid sites

To analyze positive selection of specific amino acid regions within full-length caleosin protein sequences, substitution ratios for nonsynonymous and synonymous mutation rates (dN/dS = ω, also known as *Ka*/*Ks*) were calculated. The "codeml" program of the PAML4 software package [37] was utilized to test the hypothesis of positive selection in the caleosins. The estimation of positive selection was based on the tree topology shown in Figure 2, and two pairs of models (M0/M3 and M7/M8) were selected and compared with the site-specific codeml model to test whether variable ω ratios occurred at amino acid sites. The parameter estimates and log-likelihood values for each model are provided in Table 4[39].

**Table 4:** Log-likehood values and parameter estimates of caleosins under site-specific models

**Model**	**InL**	**2∆ℓ**	**Estimates of parameters**	**Positively selected sites**
M0 (one-ratio)	-4930.58		ω = 0.12741	None
M3 (discrete)	-4866.93	127.29** (M0vsM3)	p_0_ = 0.05223, p_1_ = 0.37093 p_2_ = 0.57684, ω_0_ = 0.00000 ω_1_ = 0.06189, ω_2_ = 0.19751	None
M7 (beta)	-4855.75		p = 1.18755, q = 7.11704	Not allowed
M8 (beta&ω)	-5816.87	1922.25** (M7vsM8)	p_0_ = 0.99999, p = 0.95936 q = 1.53716, p_1_ = 0.00001 ω = 2.94912	70R** 74G** 88 L** 89G 100K** 106A 107S**

Note: p < 0.05 was marked by *p < 0.01 were marked by **;

ω: The substitution rate ratios of non-synonymous (dN or Ka) versus synonymous (dS or Ks) mutations.

Codon (amino acid) positions presented above are based on the *AtCLO*5 gene.

In the first model pair (M0/M3), the discrete model (M3) was significantly better than the one-ratio model (M0), with likelihood-rate test statistic 2∆ℓ = 127.29, which greatly exceeded the critical value of 13.28 with df = 4 (p < 0.01). Thus, M0 was rejected, indicating extreme variation in selection pressure among amino acid sites. This analysis suggested that the caleosin sequences were under positive evolutionary selection. We performed additional tests using the M7 (beta) and M8 (beta and ω) models to detect amino acid sites that were under adaptive evolution. The likelihood rate test statistic 2∆ℓ = 1922.25 greatly exceeded the critical value, and seven candidate amino acid sites for positive selection were identified (70R**, 74G**, 88 L**, 89G, 100 K**, 106A, 107S**) based on BEB analysis of M8 (**, significant at p < 0.01).We observed relationships between amino acid sites under positive selection and functional divergence (Figure 2). Sites 70R, 88 L, and 106A were under positive selection as well as Type-I and Type-II functional divergence; Sites 89G, 100 K, and 107S were under both positive selection and Type-1 functional divergence. Additionally, the seven sites were also compared to the important motifs; two sites was identified in the proline-knot motif, the other 5 sites were all located in the C-terminal domain (Figure 3).

### Coevolution of caleosin amino acid sites

Testing coevolution between sites is an essential complement to molecular-selection analysis, since methods designed to detect adaptive evolution based on Bayesian approaches do not account for evolutionary interdependence between protein residues, which would providing more biologically realistic results. We used coevolution analysis using protein sequences (CAPS), which is significantly more sensitive than other methods, to analyze coevolution in caleosins [40]. Identification of coevolved residues can provide insight into pairs of amino acid sites within a protein whose evolution is linked by structural, functional, or interacting constraints.

Three groups of amino acid sites (groups 1–3) were predicted by their hydrophobicity and molecular weight to spatially contact each other and to have coevolutionary significance. The largest was group 2, which consisted of five sites (23H, 26 F, 28D, 30 N, and 32D); groups 1 and 3 contained only two sites: 17Y and 18 N (group 1) and 161D and 176D (group 3). Comparison of the sites to the important motifs revealed that the five group-2 sites coincided with the Ca^2+^-binding motif, which was highly important to caleosin (Figure 3).

## Discussion

### Evolution of the caleosin gene family

In this study, we identified 84 caleosin genes from 15 species representing 5 main plant lineages by comparative genomic analysis. The caleosin genome sequence provides a large amount of data that can be used to explore functional diversity from multiple perspectives. A N-J phylogenetic tree including 84 distinct protein sequences clearly demonstrated that these genes could be divided into five subfamilies (Figure 1A). A ML phylogenetic tree was also constructed, which showed similar topology to the N-J tree with only minor modifications. This classification was further supported by the results of motif and exon/intron analyses. Hanano et al. proposed a classification based on evolutionary relationship in which the caleosin genes were divided into three distinct classes: class I, class II, and class III, which coincided with sub IV, sub III, and sub V, respectively [17]. Moreover, in our research we identified anther two new subfamilies in the present study: sub I and sub II, the members of which members are monocots (with two exceptions) and are predicted to have divided from the other subfamilies after the monocot-dicot split according to the proposed evolutionary pattern of caleosin. Previous research has detected a caleosin in lily pollen oil bodies that appears to be a unique isoform distinct from that in lily seed oil bodies [15]. Sequence alignment and phylogenetic tree analysis have indicated that this lily pollen caleosin and a putative rice pollen caleosin may represent a class distinct from the caleosin found in seed oil bodies. By searching the accession number of the putative rice pollen caleosin used in this previous study, we determined that it exactly matched the *OsCLO3* employed in our research, which was classified into sub II. Using BLASTP we determined that proteins in other species that were most similar to the lily pollen caleosin were all classified into sub II (*BdCLO3, SiCLO7, ZmCLO1,* and *SbCLO2*). In light of these results, we hypothesized that the distinct class in pollen discovered by previous research may belong to the same subfamily as the sub II detected in this study. Further work is required to determine if the other members of sub II are also expressed in pollen.

Segmental duplication, tandem duplication, and transposition events such as retro- and replicative transposition are the three main forces that drive the expansion of gene families [41,42]. Transposition events are difficult to identify based on sequence analysis; therefore, we focused on segmental and tandem duplication events (Tables 1 and 2). In this paper, the clustering distribution of caleosin genes (Additional file 7) revealed that tandem duplication played an additional role in determining the current size of the caleosin gene family in monocots, while segmental duplication is likely to have played a pivotal role in caleosin gene expansion in dicots. Moreover, age estimation of the *OsCLO* and *GmCLO* genes indicated that divergence within caleosin gene pairs occurred during the period of large-scale duplication events (Table 1). These observations suggest that large-scale duplication may also have been involved in the expansion of the caleosin gene family in rice and soybean.

The above analysis reveals that the caleosin gene family originated from a common ancestor, followed by lineage-specific expansion and divergence in each lineage and species during its evolution. Moreover, lineage-specific expansion, primarily by tandem duplication in monocots and by segmental duplication in dicots, is likely to have contributed to the size of the caleosin gene family. Large-scale duplication may also have been involved in the expansion of the caleosin gene family in rice and soybean.

### The identification of Motif 6

We identified 6 motifs in the present work, and the distribution of Motif 6 was strongly related with the classification of caleosin genes into subfamilies. Motif 6 was found in all members of subs II, IV, and V, with only one exception (probably due to the software parameter settings). Conversely, none of the sub I or sub III members contained Motif 6. Furthermore, the multiple alignments of sequences from the same species groups shown in Figure 3, and Additional file 5 and Additional file 6 indicated that the presence of Motif 6 played an important role in the classification of caleosins.

Previous researches have reported that rice embryo caleosin possesses an additional N-terminal appendix of approximately 40–70 residues [11,43,44]. Chen et al. found that the molecular mass of caleosin in the oil bodies of rice embryos is 32 kDa with an N-terminal appendix of 42 residues, as compared with the 27 kDa sesame caleosin. The sequence alignment of rice, adlay, maize, barley, sesame, and soybean in that study indicates that caleosins from monocot species possess an N-terminal appendix of 40–70 residues absent in those from dicot species. By searching the accession numbers of the sequences aligned in the previous research, we identified that the sequences for rice, maize, and soybean were *OsCLO4*, *ZmCLO8*, and *GmCLO5*, respectively. The sites, locations, and lengths of the N-terminal appendix identified for rice and maize in the previous work were similar to those of motif 6 in our research. However, we detected the presence of motif 6 in *GmCLO5* (which was not previously found); this result may have been due to the longer sequence of *GmCLO5* employed in the present study (50 residues more than the previous research) and the use of 84 alignment sequences (as opposed to only six in the previous work). We therefore hypothesize that motif 6 coincides with the previously discovered N-terminal appendix. On the basis of the alignment of six sequences, Chen et al. (2012) speculated that caleosins in monocot seed oil bodies possess an additional N-terminal appendix and are thus larger than those in dicot seed oil bodies. In our research, we observed that the monocot caleosins in sub I did not contain motif 6, while those in sub II and sub IV all possessed the motif. Likewise, for dicot caleosins, members of sub III did not contain motif 6, while those in sub IV all contained the motif (with one exception). Concerning the evolutionary pattern of caleosin based on the phylogenetic analysis, we suggest that the absence of motif 6 in sub I and sub III may have been the consequence of an evolutionary deletion occurring after the divergence of monocots and dicots. It remains unclear how the highly conserved caleosin isoforms execute several diverse functions that may require a well-structured active site for enzymatic reaction and a well-featured binding surface for specific protein–protein interaction. We speculate that motif 6 may be related to the diverse functions of caleosins, but this hypothesis requires further investigation.

### Functional divergence and positive selection in the caleosin gene family

Type-I and Type-II functional divergence between gene clusters of caleosin subfamilies were estimated by DIVERGE analysis [38,45]. Type-I functional divergence results in site-specific rate shifts, a typical example of which is an amino acid residue that is highly conserved in a subset of homologous genes and highly variable in a different subset of those genes [38,46]. Type-II functional divergence results in changes in cluster-specific amino acid properties [47,48]. Such divergence is exemplified by radical shifts such as positive versus negative charge differences at a homologous site that is otherwise evolutionally conserved between subfamilies within a phylogeny [49]. Significant differences in Type-I functional divergence between subfamily pairs indicated that different site-specific shifts in evolutionary rate may have occurred at certain amino acid sites, while observed Type-II divergence indicated a radical shift in amino acid properties. The analysis showed that functional divergence are mainly between subs I/IV, II/III, II/IV, and II/V. Among them, sub II seems to have been diverged in function with both subs III, IV, and V. The results are also consistent with the conjecture discussed in the above that members of sub II might belong to the same subfamily with the pollen caleosins, thus caleosins in pollen might have diverged in function with that in seed oil bodies. Moreover, the critical amino acid sites of Type-I and Type-II functional divergence may be related to the specific function of caleosin class in pollen which need further study.Positive selection has been reported to be associated with gene duplication and functional divergence. To explore whether positive selection drove the evolution of the caleosin gene family and whether selective constraints affected caleosin genes after duplication, it must be determined whether the rate of relaxation accelerated and if any amino acid residues were under positive selection. The results of positive selection analysis provided evidence for adaptive evolution, and seven sites (70R, 74G, 88 L, 89G, 100 K, 106A, 107S) were predicted to have undergone positive selection. Interestingly, six of the seven sites (all but 74G) were also identified to have been under Type-I functional divergence, while 70R, 88 L, 106A were under positive selection and under Type-I and Type-II functional divergence (Figure 2). Gene duplication may result in altered functional constraints between the gene clusters of a gene family. The results of the functional divergence analysis suggested that caleosin genes should be significantly functionally divergent from each other, especially with respect to the three amino acid residues (70R, 88 L and 106A) identified by both PAML 4 and DIVERGE 2.0 analyses, which were inferred to have played important roles during evolution.

### Coevolution in the caleosin gene family

Protein evolution depends on intramolecular coevolutionary networks, the complexity of which is proportional to the underlying functional and structural interactions among sites [40]. The more complex the coevolutionary network is for a particular site, the greater the selection coefficient may be against a mutation at that site due to the dramatic effect that this mutation would have on other coevolving protein regions. Testing for coevolution between sites is thus an essential step to complement molecular selection analysis and to provide more biologically realistic results.

Coevolution analysis of caleosin proteins detected three groups of amino acid sites. Interestingly, further analysis determined that the five sites of group 2 were distributed in the Ca^2+^-binding motif (Figure 3). The calcium-binding capacity of caleosins has been demonstrated by Takahashi et al. [50] for *AtCLO3* (encoded by *AT2G33380*) isolated from *A. thaliana* and by Chen et al. [4] for caleosin from *Sesamum indicum*. Calcium ions are capable of promoting aggregation of purified OBs and are predicted to play an important role in OB aggregation and regulation during biogenesis processes [8]. This observation suggests that during the evolution of caleosin, sites in the Ca^2+^-binding motif appear to have coevolved. Moreover, the two group-3 sites, 161D and 176D, were separated in both primary and tertiary structure. Gloor et al. [51] showed that coevolution between distant sites occurred in sites proximal to regions with critical functions, where coevolution maintained the structural characteristics of these regions and consequently maintained protein conformational and functional stability. Hence, the coevolution between 161D and 176D might contribute to maintaining the structural characteristics of caleosin.

### Critical amino acid sites of caleosin

The predicted sites of functional divergence, positive selection, and coevolution detected by bioinformatics software (DIVERGE, PAML, and CAPS, respectively) are all labeled in Figure 3. Moreover, the positions of a calcium-binding motif, a proline-knot like motif, and four phosphorylation sites (one tyrosine kinase and three casein kinase II phosphorylation sites) are indicated at the tops of the sequences based on the proposed secondary structure from Chen et al. [11]. The two proline positions of the proline-knot motif were both indicated as functional divergence sites. Among the 34 positions behind the proline-knot motif, 32 (94.12 %) were considered as functional divergence sites. Including another four sites in the proline-knot motif, 36 of 38 (94.74 %) sites were functionally divergent, and all seven sites under positive selection were also located in this region. Interestingly, the analysis showed that the critical amino acid sites of functional divergence and positive selection were mainly localized in the C-terminal domain of caleosin, while coevolutionary sites were concentrated in the calcium-binding region (Figure 3).

Both the structural stability and thermostability of artificial oil bodies have been shown to be slightly or severely reduced under the truncation of the amphipathic α-helix (15 residues) or proline-knot subdomain (21 residues) of recombinant caleosin, and the whole central hydrophobic domain of 36 (15 + 21) residues is thus crucial for the stability of oil bodies [12,52,53]. The predicted α-helix and proline-knot are labeled in Figure 3, along with the two predicted functional divergence sites and four positive selection sites coinciding with this region. A disulfide bridge between two cysteine residues (C221 and C230 of *AtCLO1*) reported by Purktova et al. is also shown in Figure 3[12]. That previous work determined that the two cysteine sites are the only cysteine residues in the caleosin primary structure and that C221 is highly conserved in all known caleosin sequences, while C230 is not as strictly conserved. However, in our alignment of eight caleosins from *A. thaliana*, we observed that this situation existed in only four members (*AtCLO1, AtCLO2, AtCLO3,* and *AtCLO6*) of sub IV (Figure 3). The cysteine sites of the caleosins in sub III are scattered across other locations. The distinct difference at crucial amino acid residues between subfamilies might contribute to answer the puzzle of how the highly conserved caleosin isoforms execute several diverse functions. Moreover, a wider physiological role for caleosins in seeds was suggested by the demonstration that the *Arabidopsis* oil-body associated *AtCLO1* protein has a calcium-dependent haem-oxygenase activity that is regulated by one or two conserved ferric-binding histidine residues 70-His and 138-His [17]. In Figure 3, 70-His and 138-His are highlighted by blue frames. The 70-His site is included in the calcium binding EF-hand motif and is also under coevolution with other sites around this motif, while the 138-His site is predicted to have undergone functional divergence during evolution. Both of these sites appear to be crucial to caleosin, but their exact roles require further clarification. All the predicted critical amino acid sites should give a firm basis for forthcoming studies of caleosin.

## Conclusion

This study provides a comparative genomic analysis addressing the phylogenetic relationships and evolution of the caleosin gene family in 15 species representing five major lineages. The results of the phylogenetic analysis revealed that five well-conserved subfamilies exist in plants and that all caleosins may have originated from a common ancestor of green plants. Among these subfamilies, sub II coincides with the distinct P-caleosin isoform recently identified in the pollen oil bodies of lily. Motif analysis detected one motif, designated as motif 6, coincident with the N-terminal appendix discovered by previous research, which may be responsible for the classification of caleosins. Tandem duplication was likely to have been the dominant mechanism of gene amplification during the expansion of the caleosin family in monocots, while the dicot caleosin family probably expanded primarily through segmental duplication. Functional divergence analysis suggested that significant site-specific selective constraints may have acted on many caleosin genes after gene duplication, leading to subfamily-specific functional evolution, and functional divergence may reflect the existence of long-term selective pressures. Further analysis showed that the majority of the detected critical amino acid sites for functional divergence and positive selection were located in the C-terminal domain of caleosin. Finally, coevolutionary analysis highlighted that the majority of detected coevolving sites were located in the Ca^2+^-binding region. The data obtained from our investigation will contribute to an improved understanding of the complexity of the caleosin gene family and of its function and evolution in green plants.

## Methods

### Identification of caleosin genes

Caleosin gene families were identified from 15 completely sequenced genomes representing the plant lineage from unicellular green algae to multicellular plants. The search was performed using "caleosin" as a keyword in the Phytozome ( http://www.phytozome.org) database; eight *Arabidopsis thaliana* caleosin genes were first retrieved and then used as a query sequence in BLAST searches (BLASTP and TBLASTN) and the sequences retrieved from corresponding plant-genome annotation resources were analyzed. Partial and redundant sequences were excluded. Sequences were obtained from the following groups and species: the unicellular green algae *Chlamydomonas reinhardtii* and *Volvox carteri* ( http://www.phytozome.org); the moss *Physcomitrella patens* ( http://genome.jgi-psf.org/); the lycophyte *Selaginella moellendorffii* ( http://genome.jgi-psf.org/); the monocotyledonous angiosperms *Brachypodium distachyon* ( http://www.brachypodium.org), *Oryza sativa* ( http://rapdb.dna.affrc.go.jp/), *Setaria italica* ( http://www.phytozome.org), *Zea mays* ( http://www.maizesequence.org), and *Sorghum bicolor* ( http://genome.jgi-psf.org/); and the dicotyledonous angiosperms *Arabidopsis thaliana* ( http://www.arabidopsis.org/), *Citrus sinensis* ( http://www.phytozome.org), *Cucumis sativus* ( http://genome.jgi-psf.org/), *Brassica rapa* ( http://brassicadb.org/brad/), *Glycine max* ( http://genome.jgi-psf.org/soybean), and *Populus trichocarpa* ( http://genome.jgi-psf.org/). In addition, the Pfam ( http://pfam.sanger.ac.uk/) and SMART ( http://smart.embl-heidelberg.de/) databases were employed to detect conserved domains with caleosin or caleosin-like protein candidates. Finally, we manually refined the search results based on the Pfam (PF05042) and SMART analyses to further reduce hits with partially conserved functional domains and other false positives.

### Phylogenetic analysis

Multiple sequence alignment was the first step in the phylogenetic analysis; alignment quality may have a significant impact on the final phylogenetic tree [54,55]. Amino acid sequences of caleosin genes were aligned using Clustal X [22,56] with the default parameters, excluding poorly aligned positions, gap positions, and divergent regions. Phylogenetic analyses were performed with a neighbor-joining (N-J) method using MEGA5 [23] and the reliability of interior branches was assessed with 1000-bootstrap resampling. To confirm the tree topologies, maximum likelihood (ML) and Bayesian trees were constructed using PhyML 3.0 [57] and MrBayes 3 [58,59], respectively, the results of which showed similar topology with only minor modifications at deep nodes (data not shown). Finally, the N-J phylogenetic tree was determined with sub V as the root (Figure 1A).

### Exon-intron structure and motif analysis

Diagrams of exon-intron structure were obtained using the online Gene Structure Display Server (GSDS: http://gsds.cbi.pku.edu.cn) with coding sequence (CDS) and genomic sequence [24]. The MEME program ( http://meme.sdsc.edu) [25,60] was used to identify motifs in the candidate caleosin protein sequences. MEME was run locally with the following parameters: number of repetitions = zero or one, maximum number of motifs = 6, and optimum motif width constrained between 6 and 50 residues.

### Estimating the age of duplicated paralog gene pairs

Pairwise alignment was performed to calculate the age of segmentally duplicated caleosin pairs using Clustal X [22]. The duplication age was estimated by the number of synonymous substitutions per synonymous site (*Ks*). The *Ks* values of the duplicated caleosin gene pairs were obtained using K-Estimator [61]. Based on 6.5 × 10^-9^ synonymous substitutions per year (λ) for rice [62], 6.1 × 10^-9^ for soybean [63], 1.5 × 10^-8^ for *Arabidopsis*[64], 9.1 × 10^-9^ for poplar [65], and 1.4 × 10^-8^ for *Brassica*[66]. The approximate age (T) of duplication events was calculated for the caleosin gene pairs using the estimated Ks values as T = Ks/2λ [67].

Fourfold synonymous third-codon transversion (D_4DTv_) distance was calculated to assess the genetic distance between tandem duplicated pairs. The paralogous proteins for each species were aligned pairwise using Clustal X [22], and corresponding codon alignments were created using the online program PAL2NAL ( http://www.bork.embl.de/pal2nal/) [68]. The corresponding codons were extracted from these alignments and used to calculate the D_4DTv_ distance between each aligning pair. D_4DTv_ values ranged from zero for recently duplicated peptides to ~0.5 for paralogs with an ancient evolutionary past.

### Functional divergence analysis

To estimate the level of functional divergence and to predict important amino acid residues among caleosin subfamilies, the coefficients of Type-I and Type-II functional divergence (θ_I_ and θ_II_) between any two clusters were calculated using the methods suggested by Gu [45,49] as implemented in DIVERGE2 [38]. This method is based on maximum-likelihood procedures to estimate significant changes in the site-specific shift of evolutionary rate or amino acid properties after the emergence of two paralogous sequences. Type-I divergence designates amino acid configurations that are very conserved in cluster 1 but highly variable in cluster 2, or vice versa, implying that these residues have experienced altered functional constraints [48]. Type-II divergence designates amino acid configurations that are very conserved in both genes but whose biochemical properties are very different, implying that these residues may be responsible for functional specification [47]. Values of θ_I_ or θ_II_ that are significantly >0 indicate site-specific altered selective constraints or a radical shift in amino acid physiochemical properties after gene duplication and/or speciation [38,47]. A site-specific profile based on posterior probability (*Qk*) was used to predict critical amino acid residues that were responsible for functional divergence. In this analysis, large *Qk* values indicated a high possibility that the functional constraint (or evolutionary rate) and/or the radical change in a site's amino acid properties differed between two clusters [38].

### Estimating the pattern of nucleotide substitution and positive-selection sites

The nonsynonymous to synonymous substitution ratio (dN/dS) for orthologous groups was computed by "codeml" in PAML [69]. The one-ratio model (M0) assumes one ω (dN, nonsynonymous/dS, synonymous) ratio for all sites. In the discrete model (M3), the probabilities (p0, p1, and p2) of each site being subjected to purifying, neutral, and positive selection, respectively, and their corresponding ω ratios (ω0, ω1, and ω2) were inferred from the data. The Beta (β) model (M7) is a null test for positive selection assuming a β distribution with ω between 0 and 1. Finally, the β and ω model (M8) adds one extra class with the same ratio, ω1 [70]. In this study, two pairs of site models were chosen in PAML to test positive selection. Analyses of real data and computer simulations [71,72] suggested that two pairs of site models were particularly effective [37]; the first pair of models was M0 (one-ratio) and M3 (discrete); the second pair was M7 (β) and M8 (β and ω). Subsequent likelihood rate comparisons were performed for M0 with M3 and M7 with M8 to determine which models better fit the data. The difference in log-likelihood between the models was compared with a chi-square distribution with *n* degrees of freedom, where *n* was the difference between the numbers of parameters in the two models. A significantly higher likelihood of the alterative model compared to the null model suggested positive selection. Finally, the Bayes Empirical Bayes (BEB) approach was used to calculate the posterior probability that each site belonged to the site class of positive selection under each model [73].

### Analysis of caleosin coevolution

To identify coevolution between amino acid sites, a Coevolution Analysis using Protein Sequences (CAPS) was performed with PERL-based software [74]. CAPS provides a mathematically simple and computationally feasible means of comparing the correlated variance of evolutionary rates at two amino acid sites corrected by time since divergence of the protein sequences to which they belong. Blosum-corrected amino acid distance was used to identify amino acid covariation. The phylogenetic sequence relationships were used to remove phylogenetic and stochastic dependencies between sites. The 3D protein structure was used to identify the nature of the dependency between coevolving amino acid sites.

## Competing interests

The authors declare that they have no competing interests.

## Authors’ contributions

WS carried out the bioinformatics, and drafted the manuscript. YH designed the study and helped to draft and edit the manuscript. YQ and YZ participated in the study and helped to draft the manuscript. GY, NW and YL coordinated the study and elaborated on manuscript. All authors read and approved the final manuscript.

## Supplementary Material

Additional file 1 **Numbers of caleosin genes identified for each species of five plant groups.** Vc, *Volvox carteri*; Cr, *Chlamydomonas reinhardtii*; Pp, *Physcomitrella patens*; Sm, *Selaginella moellendorffii*; Bd, *Brachypodium distachyon*; Os, *Oryza sativa*; Si, *Setaria italica*; Zm, *Zea mays*; Sb, *Sorghum bicolor*; Cxs, *Citrus* x *sinensis*; Br, *Brassica rapa*; At, *Arabidopsis thaliana*; Cs, *Cucumis sativus*; Gm, *Glycine max*; Pt, *Populus trichocarpa*. Click here for file

Additional file 2 Caleosin gene family in fifteen species. Click here for file

Additional file 3 Neighbor-joining (N-J) phylogenetic tree of caleosin gene family. Click here for file

Additional file 4 Motifs found in caleosins. Click here for file

Additional file 5 **Sequences alignment for each species of dicots.** Members belong to sub III and sub IV are colored in yellow and blue, respectively. The positions of Motif 6 are all labeled in the figure. Click here for file

Additional file 6 **Sequences alignment for each species of monocots.** Members belong to sub I, sub II and sub IV are colored in red, green and blue, respectively. The positions of Motif 6 are all labeled in the figure. Click here for file

Additional file 7 Segmental and tandem duplications of caleosin gene family. Click here for file

Additional file 8 Amino acid sites of functional divergence between groups of the caleosin subfamily. Click here for file
