# Supplementary figures and images for: Delineation of plant caleosin residues critical for functional divergence, positive selection and coevolution
Source: BMC Evol Biol. 2014 Jun 9;14:124. doi: 10.1186/1471-2148-14-124 (PMC4057654; doi:10.1186/1471-2148-14-124)

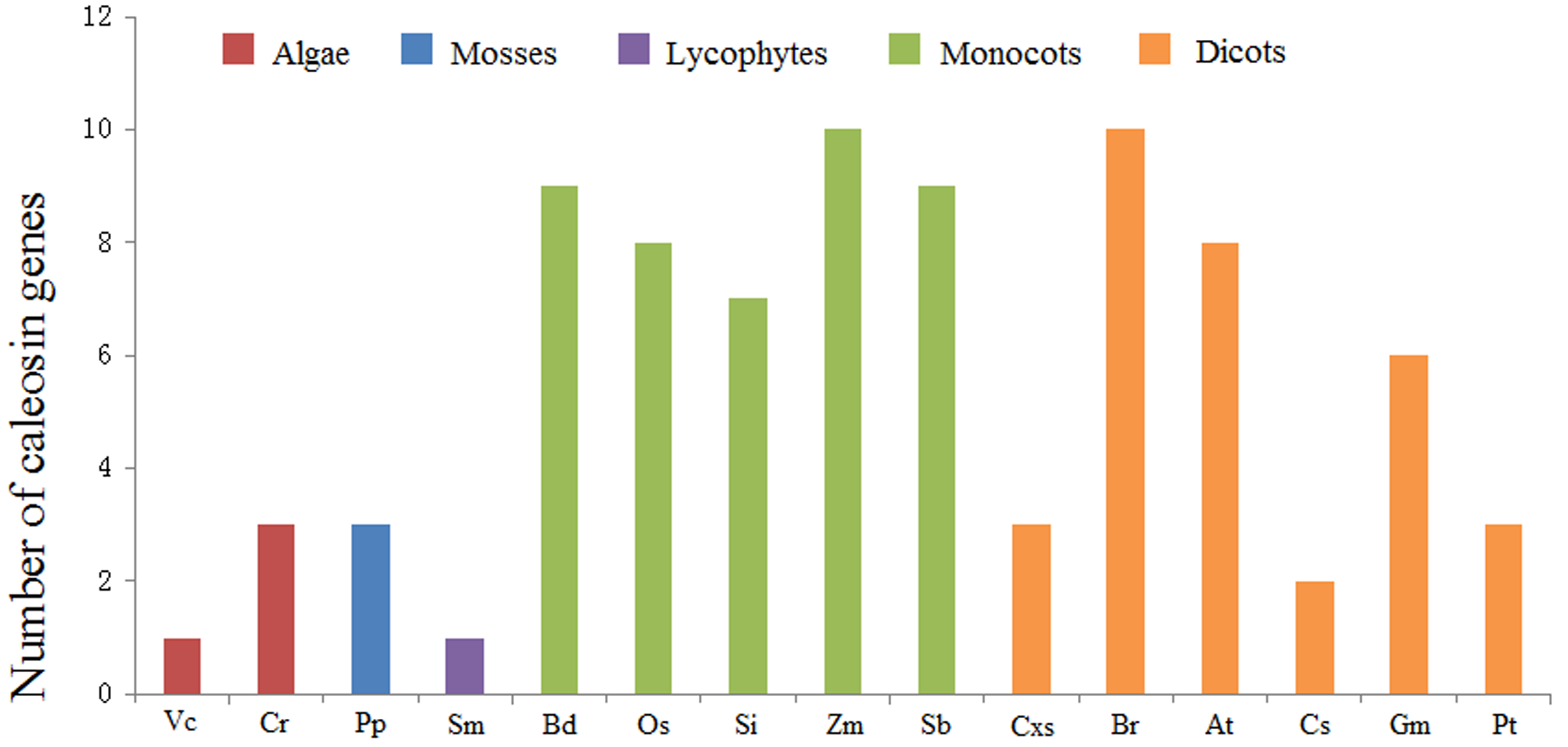

Supplement: Additional file 1 — Numbers of caleosin genes identified for each species of five plant groups. Vc, Volvox carteri; Cr, Chlamydomonas reinhardtii; Pp, Physcomitrella patens; Sm, Selaginella moellendorffii; Bd, Brachypodium distachyon; Os, Oryza sativa; Si, Setaria italica; Zm, Zea mays; Sb, Sorghum bicolor; Cxs, Citrus x sinensis; Br, Brassica rapa; At, Arabidopsis thaliana; Cs, Cucumis sativus; Gm, Glycine max; Pt, Populus trichocarpa. [file 1471-2148-14-124-S1.tiff]

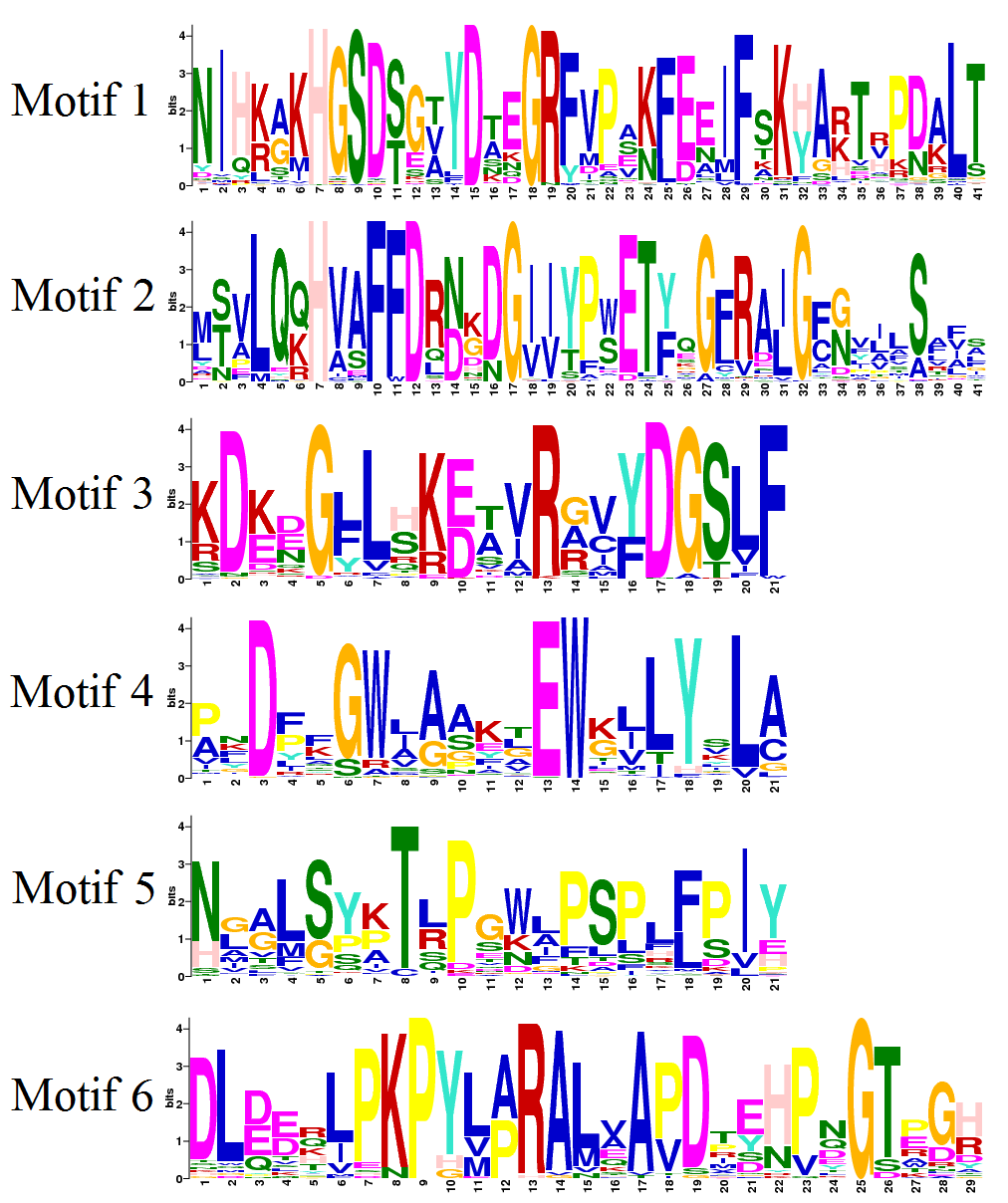

Supplement: Additional file 4 — Motifs found in caleosins. [file 1471-2148-14-124-S4.png]

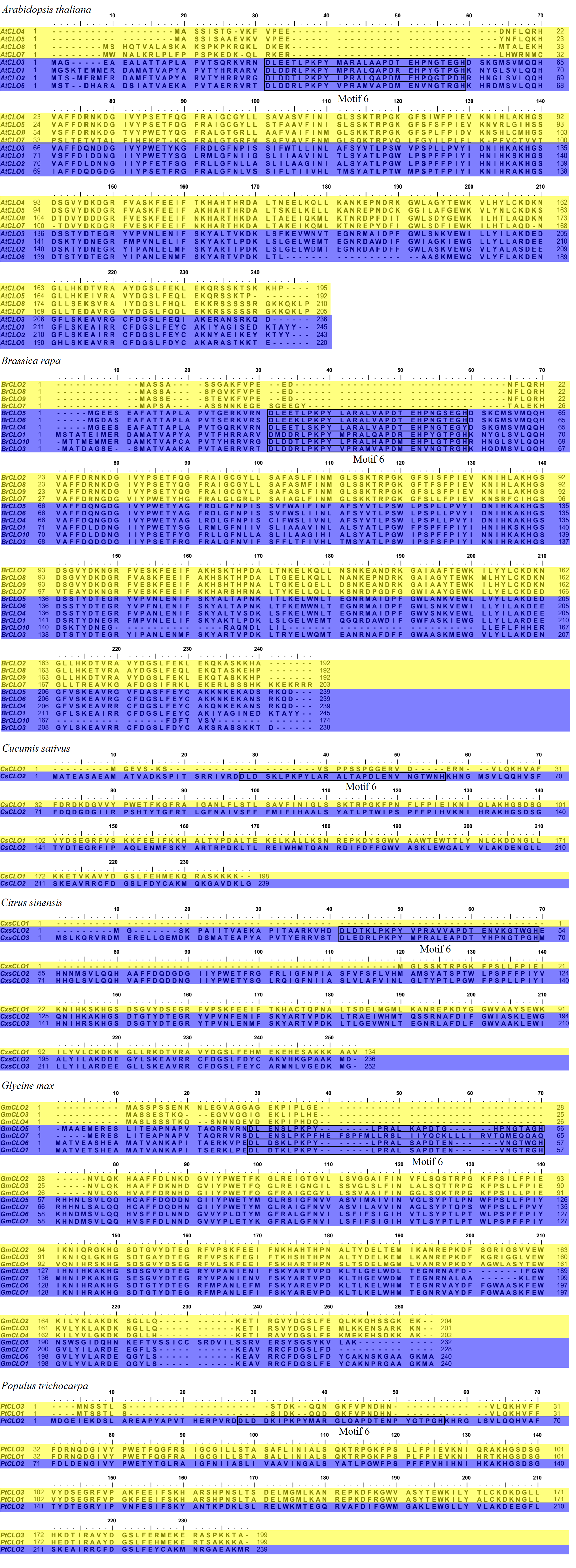

Supplement: Additional file 5 — Sequences alignment for each species of dicots. Members belong to sub III and sub IV are colored in yellow and blue, respectively. The positions of Motif 6 are all labeled in the figure. [file 1471-2148-14-124-S5.png]

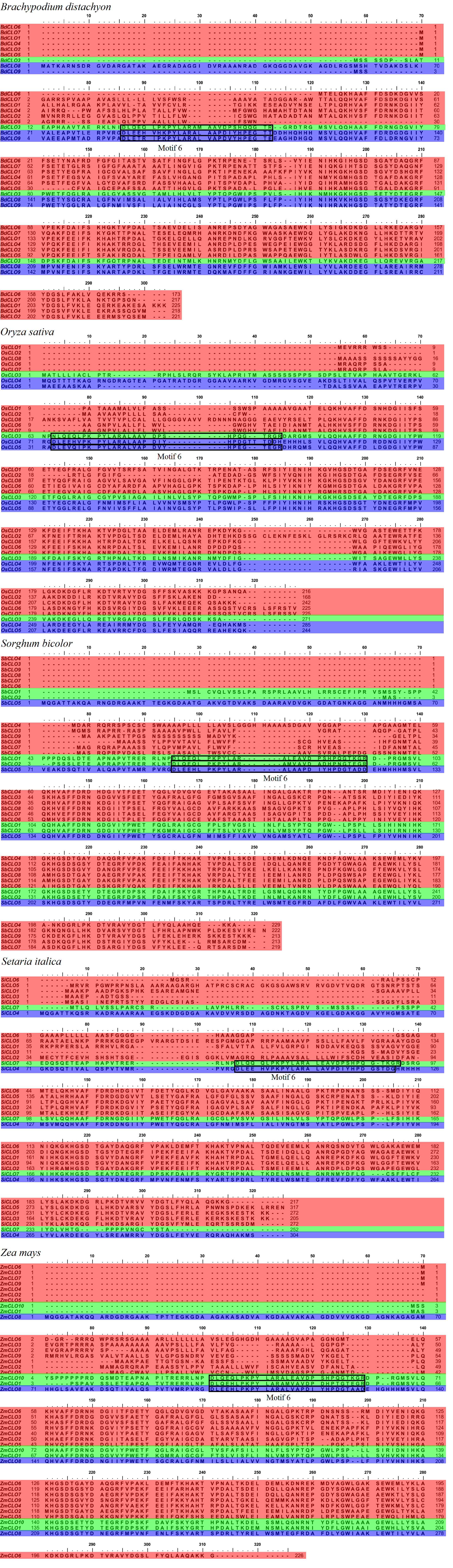

Supplement: Additional file 6 — Sequences alignment for each species of monocots. Members belong to sub I, sub II and sub IV are colored in red, green and blue, respectively. The positions of Motif 6 are all labeled in the figure. [file 1471-2148-14-124-S6.png]
