# Supplementary material for: Delineation of plant caleosin residues critical for functional divergence, positive selection and coevolution
Source: BMC Evol Biol. 2014 Jun 9;14:124. doi: 10.1186/1471-2148-14-124 (PMC4057654; doi:10.1186/1471-2148-14-124)
Supplement: Additional file 7 — Segmental and tandem duplications of caleosin gene family. [file 1471-2148-14-124-S7.docx]

| Additional file 2  Segmental and tandem duplications of caleosin gene family. | | | |
| --- | --- | --- | --- |
|  | Species | Segmentally duplicates | Tandem duplicates |
| Monocots | *Oryza sativa* | 2 | 4 |
|  | *Brachypodium distachyon* | 0 | 4 |
|  | *Sorghum bicolor* | 0 | 4 |
| Dicots | *Arabidopsis thaliana* | 4 | 4 |
|  | *Glycine max* | 4 | 2 |
|  | *Populus trichocarpa* | 2 | 0 |
|  | *Brassica rapa* | 9 | 0 |
