# Supplementary material for: Delineation of plant caleosin residues critical for functional divergence, positive selection and coevolution
Source: BMC Evol Biol. 2014 Jun 9;14:124. doi: 10.1186/1471-2148-14-124 (PMC4057654; doi:10.1186/1471-2148-14-124)
Supplement: Additional file 8 — Amino acid sites of functional divergence between groups of the caleosin subfamily. [file 1471-2148-14-124-S8.doc]

| Additional file 3  Amino acid sites of functional divergence between groups of the caleosin subfamily | | | | |
| --- | --- | --- | --- | --- |
| Group1 | Group2 | Type-Ⅰ | | Type-Ⅱ |
|  |  | Q_k_>0.8 | Q_k_>0.9 | Q_k_>0.8 |
| Ⅰ | Ⅱ | 70R, 90I |  |  |
| Ⅰ | Ⅲ |  |  |  |
| Ⅰ | Ⅳ | 70R, 71P, 75F, 80P, 81I, 82E, 83V, 84K, 87R, 88L, 89G, 90I, 91H, 92S, 93S, 94D, 96G, 97V, 98Y, 99D, 100K, 101D, 102G, 103R, 104F, 105V, 106A, 107S, 109F, 110E, 111E, 112I, 113F, 114A, 123A, 126S | 70R, 71P, 80P, 81I, 82E, 83V, 88L, 89G, 90I, 91H, 93S, 97V, 99D, 102G, 104F, 105V, 106A, 107S, 109F, 110E, 111E, 113F, 123A, 126S | 70R, 85N, 90I, 98Y, 101D, 109F, 112I |
| Ⅰ | Ⅴ | 123A | 123A |  |
| Ⅱ | Ⅲ |  |  | 70R, 83V, 88L, 90I, 97V, 98Y, 106A, 112I |
| Ⅱ | Ⅳ | 70R, 71P, 75F, 80P, 81I, 82E, 83V, 84K, 85N, 87R, 88L, 89G, 90I, 91H, 92S, 93S, 94D, 96G, 97V, 98Y, 99D, 100K, 101D, 102G, 103R, 104F, 105V, 106A, 107S, 108K, 109F, 110E, 111E, 112I, 113F, 114A, 123A, 126S | 70R, 71P, 75F, 80P, 81I, 82E, 83V, 84K, 85N, 87R, 88L, 89G, 90I, 91H, 92S, 93S, 94D, 96G, 97V, 98Y, 99D, 100K, 101D, 102G, 103R, 104F, 105V, 106A, 107S, 108K, 109F, 110E, 111E, 112I, 113F, 114A, 123A, 126S | 70R, 83V, 88L, 98Y, 108K, 109F, 112I, 123A |
| Ⅱ | Ⅴ | 70R, 71P, 75F, 80P, 81I, 82E, 83V, 84K, 85N, 87R, 88L, 89G, 90I, 91H, 92S, 93S, 94D, 96G, 97V, 98Y, 99D, 100K, 101D, 102G, 103R, 104F, 105V, 106A, 107S, 108K, 109F, 110E, 111E, 112I, 113F, 114A, 123A, 126S | 70R, 71P, 75F, 80P, 81I, 82E, 83V, 84K, 85N, 87R, 88L, 89G, 90I, 91H, 92S, 93S, 94D, 96G, 97V, 98Y, 99D, 100K, 101D, 102G, 103R, 104F, 105V, 106A, 107S, 108K, 109F, 110E, 111E, 112I, 113F, 114A, 123A, 126S | 70R, 71P, 83V, 88L, 97V, 108K |
| Ⅲ | Ⅳ |  |  |  |
| Ⅲ | Ⅴ |  |  |  |
| Ⅳ | Ⅴ |  |  |  |
